# Brown Bear, Brown Bear, What Do You See? Speakers Use More Redundant Color Adjectives When Speaking to Children Than Adults

**DOI:** 10.1162/OPMI.a.348

**Published:** 2026-04-14

**Authors:** Maya Taliaferro, Laura Schulz

**Affiliations:** New York University, New York, NY, USA; Massachusetts Institute of Technology, Cambridge, MA, USA

**Keywords:** language, communication, pragmatics, incremental collaborative efficiency hypothesis, rational speech act, development

## Abstract

Cross-culturally, the evolution and production of color words has been linked to communicative efficiency. In visual search tasks for instance, speakers will direct a listener to a target by referring to both its color and shape even when all the targets are unique. Moreover, speakers produce these redundant color adjectives more in languages where they precede the noun, like English, than in ones where they are postnominal, like Spanish. These results are consistent with an Incremental Collaborative Efficiency Hypothesis: the proposal that speakers are sensitive to listeners’ online processing demands. If this is the case, speakers should be especially likely to produce redundant color adjectives for children, since children’s visual search is especially slow. Here we show that English (Experiment 1) and Spanish-speaking (Experiment 2) adults, and parents and non-parents alike (Experiment 3), selectively generate redundant color adjectives for children. This is not because of a general bias towards being over-informative with children: when redundant color adjectives are uninformative and would not promote efficient search, speakers are no more likely to use them with children than adults (Experiment 4). Finally, children’s own use of redundant color adjectives for efficient communication changes over development. Four to ten-year-olds are less likely to produce redundant color adjectives than adults but nonetheless do so selectively when addressing younger children (Experiments 5 and 6). Collectively, these results suggest that sensitivity to listeners’ online processing demands is robust, emerges relatively early in development, and may especially benefit young learners.

## INTRODUCTION

It would be odd to start this article by informing the reader that it is a research paper on a topic of scientific interest. Leading off this way violates a basic principle of cooperative communication: people should provide only information necessary to the audience, not information that is already known or otherwise redundant (Grice, 1975). The fact that speakers try to communicate efficiently accounts for a wide range of linguistic phenomena, ranging from the fact that more predictable words are shorter to the tendency of speakers to omit words that are more probable in context (Aylett & Turk, 2004; Frank & Jaeger, 2008; Gahl & Garnsey, 2004; Jaeger, 2006; Levy & Jaeger, 2007; Piantadosi et al., 2012; Tily et al., 2009; Zipf, 1949).

However, it has long been clear that the injunction to omit unnecessary information is often honored in the breach. In referential contexts, speakers frequently provide redundant information. This phenomenon has perhaps been most extensively investigated in Visual World Paradigms (Cooper, 1974; Tanenhaus et al., 1995) in which speakers are asked to help a listener locate a target in an array. If for instance, an array consists of different colored shapes, speakers tend to mention both the shape and color of the target (e.g., “Find the blue square”) even when the shape uniquely identifies the referent. Moreover, these redundant cues turn out to be helpful to the listener, enabling them to arrive more rapidly at the referent (Arts et al., 2011; Belke & Meyer, 2002; Brown-Schmidt & Konopka, 2011; Deutsch & Pechmann, 1982; Engelhardt et al., 2006; Koolen et al., 2013; Nadig & Sedivy, 2002; Pechmann, 1989; Rubio-Fernández, 2016, 2019; Sonnenschein & Whitehurst, 1982).

Researchers have suggested that these redundancies reflect rational decisions about the tradeoffs between the cost and informativeness of communication (Degen et al., 2020), consistent with a Rational Speech Act framework. The Rational Speech Act framework formalizes the ways that speakers and listeners recursively represent each other’s beliefs and utilities to select the most informative thing to say in context (Frank & Goodman, 2012; Franke & Jäger, 2016; Goodman & Frank, 2016; see Degen, 2023 for discussion and review). Recently, researchers have suggested that one of the factors that rational speakers take into account is the incremental, online processing demands on the listener (the Incremental Collaborative Efficiency Hypothesis; Jara-Ettinger & Rubio-Fernandez, 2022; Rubio-Fernandez et al., 2021).

Some evidence for this hypothesis comes from cross-linguistic studies. If speakers are indeed sensitive to listeners’ online processing, then the position of words in a sentence, not just the words themselves, should matter. Color adjectives precede nouns in English but follow nouns in Spanish. This difference suggests that in visual search tasks, Spanish speakers should be less likely than English speakers to produce redundant color adjectives because the listener is likely to have already located the target before the color adjective is processed, and they are (Jara-Ettinger & Rubio-Fernandez, 2022; Rubio-Fernández, 2016, 2019; Rubio-Fernandez et al., 2021). By contrast, number determiners precede the noun in both languages, and English and Spanish speakers are equally likely to produce redundant number determiners (Wu & Gibson, 2021).

Children, like adults, process speech incrementally (Fernald et al., 1998, 2001, 2006; Swingley et al., 1999; Trueswell et al., 1999). However, relative to adults, children are slower at many tasks (Hale, 1990; Kail, 1986), and in particular they are delayed at visual search tasks, continuing to show improvement through late adolescence (Donnelly et al., 2007; Gerhardstein & Rovee-Collier, 2002; Hommel et al., 2004; Mackworth & Bruner, 1970; Woods et al., 2013). If speakers produce utterances that are closely attuned to listeners’ incremental, online information processing, then they might be sensitive not only to effects of language (prenominal adjectives vs. postnominal adjectives) and context (the size of the array or the uniqueness of the referent) but also to developmental differences in listeners’ processing speed.

Considerable research suggests that speakers are sensitive to both transient characteristics of the listener (what information is in common ground; Clark, 1996; Clark & Murphy, 1982; Heller et al., 2012; Horton & Keysar, 1996; Matthews et al., 2006) and stable ones (e.g., whether the listener is a native speaker or a foreigner; Papoušek & Hwang, 1991; Uther et al., 2007). Consistent with this sensitivity, people speak differently to young children than to adults in many respects (Ferguson, 1964; Papoušek et al., 1987). Child-directed speech emerges robustly across languages and cultures (Hilton et al., 2022) and has characteristic acoustic (higher pitch, elongated vowels, purer tones, etc.), and structural features (grammatical simplicity, consistency of associations between onset cues, object labels and sentence position, etc.) that facilitate language acquisition (Golinkoff et al., 2015; Hayes & Ahrens, 1988; Hills et al., 2010; Hilton et al., 2022; Ma et al., 2011; Newport et al., 1977; Snow, 1972; Yurovsky et al., 2012). Additionally, child-directed speech uses fewer words than adult-directed speech and repeats words and multiword utterances more often (Cameron-Faulkner et al., 2003; Küntay & Slobin, 2002; Soderstrom, 2007; Tal et al., 2023, 2024; Waterfall, 2006).

Some of these distinctive features of child-directed speech are rarely ever observed *except* in communication with children (Dominey & Dodane, 2004; Papoušek & Papoušek, 1987). However, as noted, redundant color adjectives are prevalent in adult-directed speech as well and visual search paradigms provide a context in which overt task demands can be equated between populations. Additionally, research suggests that prenominal modifiers (e.g., color adjectives or gendered articles) facilitate the identification of referents by age three (Fernald et al., 2010; Lew-Williams & Fernald, 2007) and that adults’ spontaneous production of both prenominal and postnominal modifiers helps children arrive at referents more quickly (Arunachalam, 2016).

Thus, we can test whether a representation of the child’s slower processing, in the mind of speakers, leads them to change their utterances in ways that selectively support incremental processing. In Experiment 1, we test the hypothesis that, controlling for task context, prosody, and syntactic cues, speakers (consciously or unconsciously) accommodate children’s slower visual search by producing more redundant color adjectives when they believe they are speaking to a child than an adult. In Experiment 2, we ask whether any tendency to adjust to children’s processing demands depends on experience with children (i.e., might parents, who are better attuned to children’s slower processing speeds be more likely to use redundant color adjectives than non-parents). In Experiment 3, we ask to what extent this holds even in postnominal languages like Spanish given that late-arriving modifiers would be more useful for Spanish-speaking child than adult listeners given children’s delayed visual search. In Experiment 4, we look at whether speakers tendency to produce redundant color adjectives for children is due to a general bias towards providing children with more information or specifically reflects a sensitivity to children’s slower online processing demands and thus occurs selectively, only when these adjectives support more efficient search.

Finally, in Experiments 5 and 6, we look at whether young children themselves are sensitive to listeners’ incremental processing demands. Children’s sensitivity to conversational pragmatics improves throughout middle childhood (Glenwright & Pexman, 2010; Grigoroglou & Papafragou, 2019; Kronmüller et al., 2014; Lagattuta et al., 2010; Lalonde & Chandler, 2002; Lecce et al., 2019; Osterhaus & Koerber, 2021; Osterhaus et al., 2016; Salomo et al., 2013). Nonetheless, preschoolers are able to adjust their communication to learners’ knowledge, goals, competence, and utilities (Bridgers et al., 2020; Dunn & Kendrick, 1982; Grigoroglou & Papafragou, 2019; Gweon & Schulz, 2019; Gweon et al., 2018; Shatz & Gelman, 1973) and they are sensitive to age differences between themselves and toddlers (Dunn & Kendrick, 1982; Magid et al., 2018; Shatz & Gelman, 1973) as well of course to differences between themselves and adults (e.g., VanderBorght & Jaswal, 2009). In Experiments 4 and 5, we look at the development of children’s sensitivity to listeners’ processing demands from ages four to ten and the extent to which children selectively use redundant color adjectives when speaking to younger children (toddlers) versus adults.

## EXPERIMENT 1

**Figure 1.**
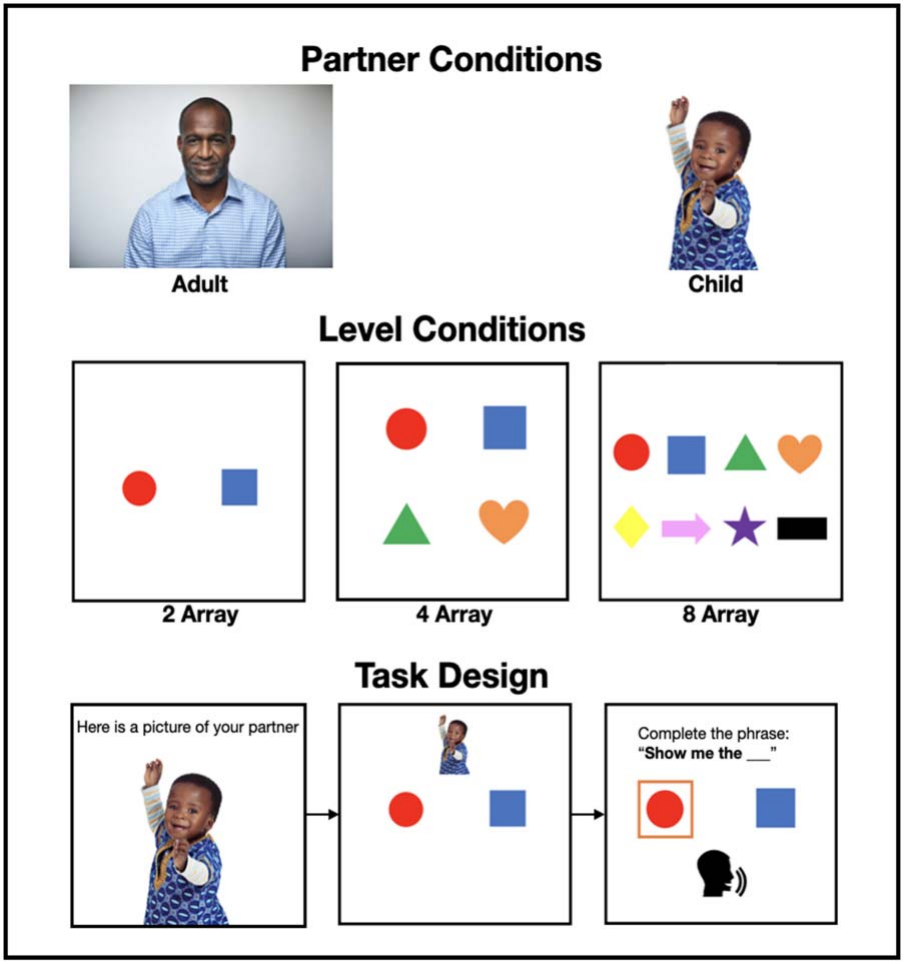
Example stimuli used in Experiments 1–3. Top row shows possible two partner conditions, second row the three level conditions and bottom row the task design.

We adapt the visual search paradigm used in previous research (Rubio-Fernández, 2016, 2019; Rubio-Fernandez et al., 2021) to look at whether adults are more likely to produce redundant adjectives when they believe they are speaking to young children than adults. Although adults believed their recordings would be used for a shape selection task with either young children or adults, there was no real audience. To remind participants of the target audience, stock images (from istockphoto.com) were used throughout (see Figure 1). We wanted the child listener’s youth to be very salient so we chose images of three-year-old children. Previous work has also shown that speakers tend to use redundant color adjectives more often as the display density increases (Jara-Ettinger & Rubio-Fernandez, 2022; Rubio-Fernandez, 2019; Rubio-Fernandez et al., 2021), thus here we compared three levels of array density—2, 4, and 8 shapes—resulting in a 2 × 3 between participants design.

### Methods

#### Participants.

Adults (*N* = 240) were recruited and tested on the online research platform Prolific.com (132 females, 108 males; mean age = 36.0, *SE* = 0.87). Inclusion required adults to be native, fluent English speakers and pass the inclusion trial below. 10 participants were excluded and replaced during recruitment due to failing inclusion criteria and 16 were excluded and replaced due to poor audio quality. The overwhelming majority of participants completed all 10 trials, with less than 5% of total trials lost.

#### Procedure.

The sample size (determined through power analysis), experimental design, inclusion criteria, and statistical models for this and all subsequent experiments were pre-registered on the Open Science Framework (OSF): https://osf.io/7fysr. Each participant was randomly assigned to one of two conditions: child partner or adult partner. Within each condition, participants were randomly assigned to a 2-shape array, 4-shape array or 8-shape array in one of three pseudo-random display orders such that there were 40 participants per array type.

Participants were given the following instructions: “In this task your goal is to help your partner identify an object in the array as fast as possible. Your partner will see the same items displayed but presented in different positions than in the array that you see. You will be generating instructions by repeating the words “Show me the ___” and completing the sentence out loud. Your instructions will be recorded and presented to your partner at the end for them to complete the task.” Participants were told that their partner would be either a child or an adult. At the start of the experiment, each participant was presented with an image of a child or adult to remind them of the age of their partner. Both images portrayed a person of the same gender and race (e.g., a Black boy and man) so that age was the only factor differentiating them.

Participants were shown an example trial and an inclusion trial. The trials began with a screen in which participants saw the array and an image reminding them of the age of their partner. Participants then clicked to the next screen. The image of the partner disappeared, the target was identified with an orange border, and participants were prompted to record their response. The example trial involved an array of three shapes: a purple star, a red circle and a green triangle; the inclusion trial involved an array of three utensils: a knife, a fork and a spoon. The spoon was the target and participants who did not record the phrase “show me the spoon” were excluded from further analysis. Then the test displays began. Every participant received ten trials. All test displays used fixed color-shape pairings: green triangle, red circle, purple star, black rectangle, yellow diamond, blue square, pink arrow, and orange heart. Across the ten trials, these shapes were presented such that each shape was the target at least once. See Figure 1.

After finishing the test trials, participants were presented with two attention-check questions. 1) Did your partner see the same shapes you saw? and 2) Did they see them in the same order? Participants who did not answer ‘yes’ for question 1 and ‘no’ for question 2 were excluded from further analysis. Data were analyzed, blind to condition, from transcripts of the audio recordings (Pilot data on 20 participants found that Phonic.ai, an integrated audio recording/transcribing service for Qualtrics, was 100% accurate at transcribing and this was used throughout). Phrases that included a color adjective were coded as a ‘1’ while phrases without a color adjective were coded as a ‘0’. This coding was agnostic to the noun label used (e.g., referring to a “ball” rather than “circle”); however, responses which did not follow the format of “show me the ___” or which did not include a specific noun label (e.g., “show me the red one”) were excluded from analysis.

### Results and Discussion

We ran a generalized mixed effects model using the glmer function as part of the lme4 package in R (Bates et al., 2015). The binary outcome variable was the presence/absence of a color adjective (1 = color adjective, 0 = bare noun) with level (display density) and condition (partner type) as predictors:

$$\text{Color}\;\text{Adj}.∼\text{level}\_\text{condition}+\text{partner}\_\text{condition}+\left(1|\text{Item}\right).$$

**Figure 2.**
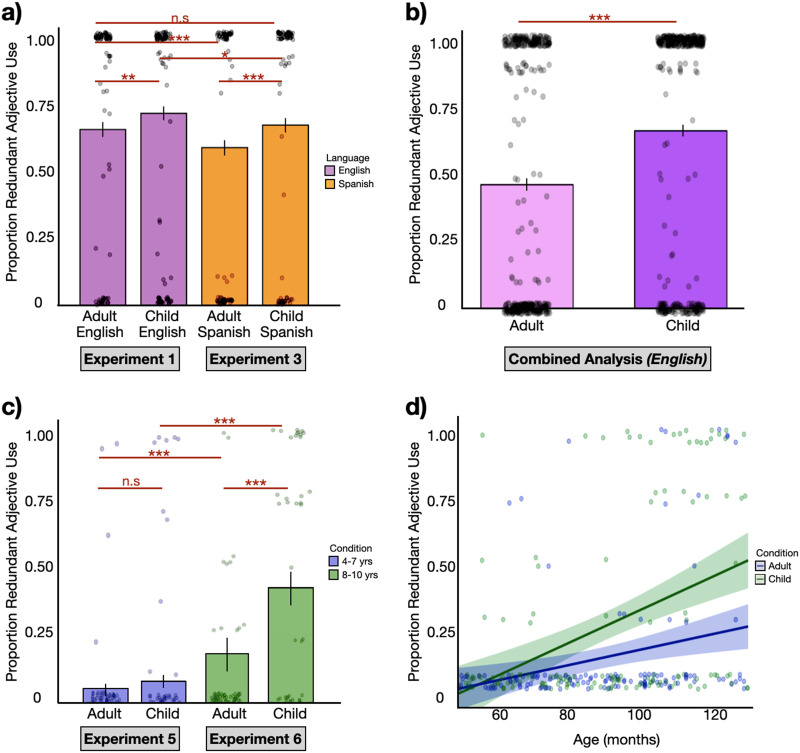
(a) Proportion of redundant color use for English speakers in Experiment 1 (purple) and Spanish speakers in Experiment 2 (orange). Black lines indicate confidence intervals. Dots represent mean adjective use per participant. (b) Proportion of redundant color use for English speakers in combined analysis collapsed across English speakers in Experiments 1 and 2 as well as additional participants. Black lines indicate confidence intervals. Dots represent mean adjective use per participant. (c) Proportion of redundant color adjective use for children in Experiment 5 (blue) and Experiment 6 (green). (d) Collapsing across Experiments 5 and 6, there was a significant effect of age on children’s tendency to produce redundant adjectives when speaking to children vs. adults. Dots represent mean adjective use per child.

As predicted, adults were more likely to use color adjectives when they believed they were speaking to a child than an adult (*β* = 0.269, *p* < 0.005. In contrast to previous work (Rubio-Fernandez, 2019, 2021) we found no effect of array density. 2 array vs. 4 array: *β* = −0.028, *p* = 0.791; 2 array vs. 8 array: *β* = 0.127, *p* = 0.238; 4 array vs. 8 array: *β* = −0.155, *p* = 0.318). See Figure 2a, purple bars.

This may be because these earlier studies were conducted in person, in a university lab, allowing standardized viewing conditions; given the variable screens used by online participants in the current study the clutter and appearance of the arrays might have differed across participants. Prior research suggests that people are sensitive to the discriminability of colors along a perceptual continuum and this affects their use of redundant color adjective (Rubio-Fernandez, 2021), and the within participant variability of the psychophysical properties of the stimulus might have obscured any condition differences. Note also that we capped our array size at 8 shapes (since a larger array would have seemed less plausible that participants were talking to three-year-olds) and participants were tested between subjects, so each participant saw only a single array size. This discrepancy could also be related to the speaker-listener interaction which exists for in lab studies, but not online. For instance, if participants saw that their listener (be that an adult or a child) is taking longer to find a target because the display is dense with shapes, they may provide more details about the target. Importantly however, although we did not replicate an effect of array density, our main prediction was confirmed: Adults were more likely to produce redundant color adjectives when they believed they were speaking children than adults.

## EXPERIMENT 2

Does speakers’ sensitivity to children’s incremental online processing depend on the speaker’s experience and familiarity with young children? While there are many ways that adults may get experience talking to young children (as a preschool teacher, as a babysitter, as an aunt or uncle, etc.), most non-parent adults have much more limited experience talking to very young children than parents do. The few previous studies that have directly compared parents and non-parents’ interactions have found considerable overlap in how parents and non-parents act towards children, but some kinds of behaviors do depend on parental experience, including attention to infant faces (Thompson-Booth et al., 2014), interventions to induce positive attitudes towards children (Jones et al., 2022), mental state attributions to infants (Shinohara & Moriguchi, 2017), and prosodic disambiguation and positive affect in child-directed speech (Kempe et al., 2010). Arguably, parents may also be more likely than non-parents to be familiar with children’s slower processing speeds, and may be more likely than non-parents to provide compensatory cues to support visual processing. Here we look at whether parents and non-parent differ in their tendency to generate redundant color adjectives for young children.

### Methods

#### Participants.

We recruited 240 English-speaking adults, 120 parents (69 females, 51 males; mean age = 32.28, *SE* = 0.60) and 120 non-parents (55 females, 65 males; mean age = 34.25, *SE* = 1.13) on Prolific.com. Because we thought any effect of parenting on adult speech would be strongest for adults currently parenting a verbal but very young child, we restricted the parent group to those with children born in 2018–2021: children who would have been between the age of two and six at the time of the study. Non-parents had to indicate that they had never raised children. Both criteria were set using Prolific screeners, ensuring the experiment was shown only to participants who qualified but that participants themselves did not know that they were being selected based on their parenting status. The inclusion criteria were otherwise as in Experiment 1. 13 participants were excluded and replaced during recruitment due to failing inclusion criteria and 9 were excluded and replaced due to poor audio quality. As with Experiments 1 and 2, less than 5% of total trials were lost.

#### Procedure.

The procedure was identical to Experiment 1 except that all participants were presented with the child as their partner. Therefore, in this study the two conditions were either being parent or non-parent rather than the child and adult conditions of the previous studies.

### Results and Discussion

We ran the same mixed effects model used in Experiment 1 and observed no effect of parenting (*β* = −0.091, *p* = 0.286) and again, no effect of array (2 array vs. 4 array: *β* = −0.069, *p* = 0.500; 2 array vs. 8 array: *β* = 0.144, *p* = 0.171; 4 array vs. 8 array: *β* = −0.214, *p* = 0.103). We cannot rule out the possibility that some of the non-parent adults had extensive experience with young children in other contexts (i.e., through other kinship relations or professionally), however, it is unlikely that this was true of most of the childless adults. Rather, these results suggest that adults in general are sensitive both to listeners’ incremental online processing and children’s delayed visual search and adapt their communication accordingly.

#### Subject Effects and Combined Analysis.

The previous studies were underpowered to include subjects as a random intercept in the model. A power analysis assuming 0.8 power and a medium effect size of 0.5 leads to 340 participants/condition to test for participant effects. We recruited 240 more adults addressing adult participants, randomly assigned to a 2-shape array, 4-shape array or 8-shape array in one of three pseudo-random display orders (as in Experiment 1). Thus, in total, we could now analyze 360 adults addressing adult participants (120 from Experiment 1 and 240 of the newly recruited participants) and 360 adults addressing children (120 from Experiment 1 and 240 collapsing across the parent and non-parent conditions of Experiment 2). We ran a *glmer*(*adj* ∼ *level* + *condition* + (1|*subj*). This model replicated the effects of Experiments 1 and 2, showing an effect of audience design such that adults were more likely to use redundant color adjectives when speaking to children than adults (*β* = 23.33, *p* < .0001) and again, no effect of array (2 array vs. 4 array, *β* = −0.288, *p* = 0.703; 2 array vs. 8 array: *β* = −0.253, *p* = 0.733; 4 array vs. 8 array: *β* = −0.039, *p* = 0.985). See Figure 2b.

## EXPERIMENT 3

As discussed, speakers of languages with postnominal modification, like Spanish, are less likely to use redundant color adjectives than those with prenominal modification, like English, possibly because postnominal adjectives are processed too late to aid visual search (Rubio-Fernández, 2016, 2019). Here we look at whether Spanish speakers, like English speakers, nonetheless produce more redundant color adjectives when they believe they are speaking to children than adults. If so, this would provide additional evidence for the Incremental Collaborative Efficiency Hypothesis: A speaker who is attuned to listener’s online processing may recognize that although the adjective will be processed relatively late in a language with postnominal modification like Spanish, it still may be processed in time to benefit a child listener with delayed visual search.

### Methods

#### Participants.

Adults (*N* = 240) were recruited on the online research platform Prolific.com (106 females, 134 males; mean age = 29.54, *SE* = 1.60). Inclusion required adults to be native, fluent Spanish speakers and pass the inclusion trial below. 17% of participants were monolingual Spanish speakers and the large majority lived outside of the USA (54% lived in Mexico, 26% in Spain, 11% in Chile and 6% in other countries). 11 participants were excluded and replaced during recruitment due to failing inclusion criteria, 17 were excluded and replaced for poor audio quality, and 1 was excluded and replaced for providing responses in English. As with Experiment 1, less than 5% of total trials were lost.

#### Procedure.

The procedure was identical to the procedure in Experiment 1 except that all materials were translated into Spanish by a native Spanish speaker. Additionally, the purple star was changed to a purple octagon so that all shapes used masculine gender pronouns in Spanish and could not be distinguished by the gender of the article preceding them.

### Results and Discussion

We ran the same mixed effects model used in Experiment 1 and observed the same pattern^1^. Spanish-speaking adults also produced more redundant adjectives when speaking to children than adults (*β* = 0.353, *p* < 0.001). Again, there was no effect of array (2 array vs. 4 array: *β* = −0.189, *p* = 0.064; 2 array vs. 8 array: *β* = −0.012, *p* = 0.910; 4 array vs. 8 array: *β* = −0.200, *p* = 0.121). See Figure 2a, orange bars. Per a pre-registered analysis, we then compared the English-speaking adults in Experiment 1 with the Spanish-speaking adults in Experiment 2. English speakers produced more redundant color adjectives than Spanish speakers in both the child condition (*t* = 2.19, *p* < 0.05) and the adult condition (*t* = 3.31, *p* < 0.001). An exploratory difference of differences (DoD) analysis between the adults and children condition in English and Spanish datasets revealed a DoD of −0.083 (*SE* = 0.12). A *z* test of the DoD did not yield statistically significant differences (*z* = −0.678, *p* = 0.49), indicating that the difference in redundant color adjective use when speaking to a child compared to an adult is the same for English and Spanish speakers.

These results provide further support for the Incremental Collaborative Efficiency Hypothesis. Speakers of languages with prenominal modification like English produce more redundant color adjectives than speakers of languages with postnominal modification, like Spanish but speakers of both languages take listener’s online processing into account and provide more redundant information to children than to adults.

## EXPERIMENT 4

The data presented so far are consistent with the hypothesis that speakers are sensitive to listeners’ online incremental processing, and thus more likely to provide redundant color adjectives to children than adults, but they do not rule out the possibility that adults simply provide more information to children than adults in general. If this is the case, then adults might be more likely to provide redundant color adjectives to children than adults, even if the redundant color adjectives do not support more efficient visual search. To investigate this possibility, in Experiment 4, we replicate the design of Experiments 1–3 but with arrays in which all the shapes are the same color. If adults habitually provide more redundant information to children than to adults, they should continue to do so in this experiment, even though the color is no longer informative. If instead, adults selectively generate redundant color adjectives to assist children’s slower incremental online processing, then in this experiment, where the redundant color adjectives provide no advantage in visual search, speakers should be no more likely to produce them for children than for adults^2^.

### Methods

#### Participants.

Adults (*N* = 120) were recruited and tested on the online research platform Prolific.com (68 females, 52 males; mean age = 43.90, *SE* = 12.52). As in Experiments 1 and 2, the inclusion criteria required adults to be native, fluent English speakers and pass the inclusion trials. Twenty-three participants were excluded and replaced during recruitment due to failing inclusion criteria and 3 were excluded and replaced due to poor audio quality. The majority of participants completed all 10 trials, with less than 5% of total trials lost.

#### Procedure.

The Procedure was identical to the Procedure in Experiment 1 except that every participant saw only the 8-shape array and within each array all the shapes were the same color: either blue, yellow, pink, red, black, purple, orange or green.

### Results and Discussion

Per our pre-registered analysis, we ran the following generalized mixed effects model using subjects as the intercept:

$$\text{Color}\;\text{Adj}.∼\text{partner}\_\text{condition}+\left(1|\text{Subject}\right).$$

**Figure 3.**
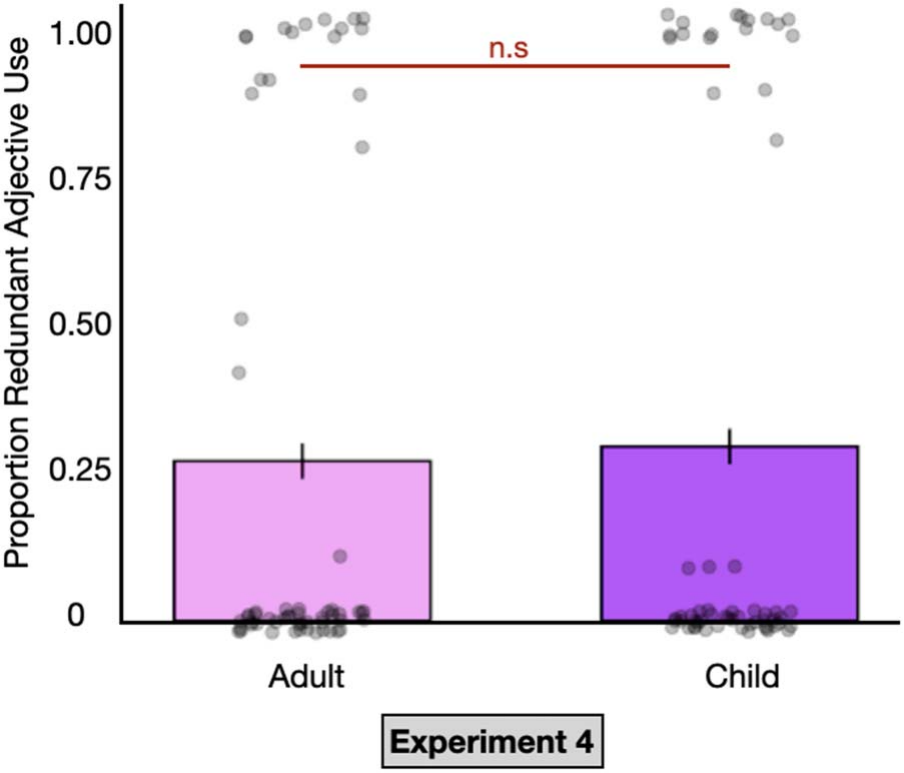
Proportion of redundant color adjectives generated by adult English speakers in Experiment 4 when speaking to adults (pink) and children (purple). Black lines indicate confidence intervals. Dots represent mean adjective use per participant.

Consistent with the online incremental processing hypothesis, there was no effect of partner condition (*β* = 0.26, *p* < 0.87). See Figure 3. This null effect suggests that redundant color adjective use is not driven by a general tendency to overspecify for children. Rather, the results are consistent with our hypothesis that speakers are more likely to provide redundant color adjectives to children than adults specifically to compensate for their slower incremental online processing and improve the efficiency of visual search.

## EXPERIMENT 5

Adults selectively provide redundant color adjectives to young children in referential search tasks; do children do so as well? Children’s ability to tailor their communication to the needs of the listener improves with age and experience (Carmiol & Vinden, 2013; Davies & Kreysa, 2018; Davis-Unger & Carlson, 2008; Matthews et al., 2007; Maynard, 2003; Strauss et al., 2002). In particular, children’s ability to understand aspects of pragmatics, including the ways that evidence can be open to different interpretations and non-literal and contrastive pragmatic inferences continue to develop through middle childhood (Glenwright & Pexman, 2010; Kronmüller et al., 2014; Lagattuta et al., 2010; Lalonde & Chandler, 2002; Lecce et al., 2019; Osterhaus & Koerber, 2021; Osterhaus et al., 2016).

Nonetheless, young children are active informants, from the earliest stages of development. Even two-year-olds selectively communicate information unknown to their audience (O’Neill, 1996), selectively correct others’ false beliefs (Knudsen & Liszkowski, 2012), and communicate verbally when pointing is ambiguous (O’Neill & Topolovec, 2001). By preschool, children are more informative when their conversational partner lacks epistemic access to a scene (Matthews et al., 2006) and can decide what information to share or withhold to effectively teach and deceive others (Rhodes et al., 2015). Children also tailor the information they provide to others’ goals and competence (Gweon & Schulz, 2019), consider others’ expected costs and rewards in deciding what to teach (Bridgers et al., 2020), and strategically inform others to manage their own reputation (Asaba & Gweon, 2022). And cross-culturally, older children (and especially siblings) teach and inform younger ones (Azmitia & Hesser, 1993; Maynard, 2003).

Of particular relevance to the current study, children adapt their communication in many respects when speaking to younger children. Most of these have looked at infant-directed speech and found that three and four-year-olds speak more slowly and use shorter utterances and less complex constructions when talking to infants rather than adults (Dunn & Kendrick, 1982; Sachs & Devin, 1976; Shatz & Gelman, 1973; Warren-Leubecker & Bohannon, 1983; Weppelman et al., 2003; Woollett, 1986). We could not use infants as listeners in the current study since no one (even a preschooler) would expect an infant to know their shapes and colors. However, children do indeed know many canonical color and shape terms by 30-months (Shatz et al., 1996; Verdine et al., 2016). Thus, although we of course did not expect four to seven-year-olds to have any specific knowledge about the development of children’s shape and color concepts, two-year-olds were a more prima facie plausible audience for the design. We tested four to seven-year-olds as speakers both because the bulk of previous work suggesting children’s flexibility as informants has focused on this age range and because, as noted, other work suggests that children as young as four distinguish age differences between themselves and two-year-olds (e.g., Magid et al., 2018). In sum, in Experiment 4, we look at whether children as young as four to seven are sensitive to listeners’ online incremental processing and provide more redundant color adjectives when they believe they are speaking to a two-year-old rather than an adult.

### Methods

#### Participants.

We recruited 160 native English-speaking children on the Children Helping Science platform: 40 four year olds (21 girls, 19 boys; mean age = 4.47 yrs, *SE* = 0.05), 40 five year olds (22 girls, 18 boys; mean age = 5.47 yrs, *SE* = 0.05), 40 six year olds (18 girls, 22 boys; mean age = 6.44 yrs, *SE* = 0.04), and 40 seven year olds (25 girls, 15 boys; mean age = 7.56 yrs, *SE* = 0.04). Half of each age bin was randomly assigned to each condition so that there were 20 children per condition. 12 participants were excluded and replaced during recruitment due to failing inclusion criteria, 28 were excluded and replaced due to poor audio quality, 4 were excluded and replaced due to parental interference, and 11 were excluded and replaced due to not completing all the trials.

#### Procedure.

Since there was no effect of array size in previous studies, we did not vary arrays in this study; every child saw an eight shape display. The image of the child was changed from previous studies to a two-year-old {Ellie} rather than a three-year-old to ensure the child appeared younger than even the four-year-old participants. The same adult image {Mr. Smith} used in previous studies was used here. The choice of toddler image was arbitrary and in contrast to the adult studies, the adult and child partner images differed in gender; however, there is no theoretical or empirical basis for assuming that this would affect children’s tendency to produce redundant color adjectives. The experiment was automated (i.e., no experimenter was present) and was administered on the online platform in families’ homes.

The experiment began with an introduction “We are going to be helping our friend today. {Ellie/Mr. Smith} is playing a game where s/he needs to find a special object. You can see the same objects s/he sees. But her/his objects can be in any order. They could look like this {an image of the objects in one order was displayed} or this {an image of the objects in a different order was displayed}, or like this {an image of the objects in a third order was displayed}. “So you can’t just point to the object, you have to tell her/him out loud! The special object {Ellie/Mr. Smith} needs to find is right here. You can see the object wiggle, but {Ellie/Mr. Smith} can’t! Your job is to get {Ellie/Mr. Smith} to find the special object as fast as possible. {Ellie is only two/Mr. Smith is very busy}, so s/he will need to be reminded what s/he is looking for by saying the phrase: show me the ___.” Participants were then shown two sample arrays with black and white images of a knife, a fork and a spoon. In this inclusion task the spoon was always indicated as the target shape and participants were told explicitly to say, “Show me the spoon”. They then were told: “Remember, you have to say: ‘show me the’ every time so {Ellie/Mr. Smith} knows you are talking to her/him.

The same inclusion task from Experiment 1 was used. Unlike in Experiment 1, target shapes for participants were identified by moving back and forth on screen and children received only four trials rather than ten. All video recordings were collected through the Children Helping Science platform and coded by individuals blind to the condition and hypothesis of the experiment.

### Results and Discussion

We ran a pre-registered mixed effects model using the glmer function in R (Bates et al., 2015). Here the binary outcome variable was the presence/absence of a color adjective with condition (partner type) and age (in years, continuous and centered) as predictors:

$$\text{Color}\;\text{Adj}.∼\text{partner}\_\text{condition}+\text{age}\_\text{centered}+\left(1|\text{Item}\right).$$

For four to seven-year-olds, we found a marginal effect of condition (*β* = 0.003, *p* = 0.055) and no effect of age (*β* = 5.23e−03, *p* = 0.502). These results provide suggestive evidence that young children are more informative when they believe they are speaking to a two-year-old than an adult. To further test if the effect of the partner condition is impacted by age, we ran a more complex interaction model as an exploratory analysis. Here the binary outcome variable was the presence/absence of a color adjective with condition (partner type) and age (in years, continuous and centered) as main effect predictors as well as interaction between condition and age:

$$\text{Color}\;\text{Adj}.∼\text{partner}\_\text{condition}*\text{age}\_\text{centered}+\left(1|\text{Item}\right).$$

Four to seven-year-olds showed a marginal effect of condition (*β* = 0.003, *p* = 0.055). There was no effect of age (*β* = 0.095, *p* = 0.7882) and no interaction between age and condition (*β* = 0.044, *p* = 0.882). These results again provide suggestive evidence that four to seven-year olds are beginning to be sensitive to younger listeners’ delayed information processing and adjust their communication accordingly. To the degree that this is the case, is it not because children are over-informative in general. Rather, as clear in Figure 2c, children of this age rarely produced redundant adjectives in either condition although they tended to do so more often for younger audiences.

Given that four to seven-year-olds are sensitive to listeners’ knowledge and competence in many aspects of communication, why did they only marginally adjust their communication for younger children? One possibility is that children in this age range did not perceive much of an age difference between themselves and the two-year-old listener. However, we think this is unlikely given that the children were explicitly told that the child listener was “only two” and prior work suggests that even the youngest children in the participant pool are sensitive to such age differences. Another possibility is that children did perceive the listener as younger than themselves but did not attribute slower visual search to the younger child, did not realize that redundant color adjectives might compensate for this, or both. Providing redundant modifiers is a relatively subtle pragmatic adjustment and the theory of mind demands might simply be beyond the reach of young children. A final possibility is that independent of any effect of listener characteristics, children in this age range might not recognize that color adjectives are valuable cues for visual search in general. Four to seven-year-olds were less likely to produce redundant color adjectives overall than adults, suggesting that children may not see them as especially informative. In Experiment 5, we try to disambiguate these accounts and further probe the development of children’s sensitivity to listeners’ online incremental processing by testing older children: eight to ten-year-olds.

## EXPERIMENT 6

As noted, the development of children’s understanding of pragmatics and ambiguity develops well through middle childhood (Glenwright & Pexman, 2010; Kronmüller et al., 2014; Lagattuta et al., 2010; Lalonde & Chandler, 2002; Lecce et al., 2019; Osterhaus & Koerber, 2021; Osterhaus et al., 2016), and it is likely that children’s understanding of other children as less capable than themselves may depend on the age difference between themselves as younger children. In Experiment 5 we replicate Experiment 4 with older children, ages 8–10, to see if they are more likely to modify their communication depending on whether they believe they are speaking to a much younger child (age two) or an adult.

### Methods

#### Participants.

We recruited 120 native English-speaking children: 40 eight year olds (17 girls, mean age = 8.55, *SE* = 0.04), 40 nine year olds (16 girls, mean age = 9.44, *SE* = 0.04), 40 ten year olds (18 girls, mean age = 10.54, *SE* = 0.04), in age bins, with half of each age group randomly assigned to each condition such that there were 20 children/condition. 8 participants were excluded and replaced during recruitment due to failing inclusion criteria 23 were excluded and replaced due to poor audio quality, 1 was excluded due to having answers provided by parents, and 6 were excluded and replaced due to incomplete data.

#### Procedure.

The procedure was identical to the procedure in Experiment 5.

### Results and Discussion

We ran the same mixed effects models used in Experiment 5. For the simplest model, we find a significant effect of partner condition (*β* = 0.225, *p* < 0.001) and a significant effect of age (*β* = 0.087, *p* < 0.001) and in the more complex model there was a significant effect of partner condition (*β* = 0.009, *p* < 0.001), a significant effect of age (*β* = 0.003, *p* < 0.001), and a significant interaction (*β* = 0.004, *p* < 0.001). Older children provide more redundant color adjectives for two-year-olds and this effect is modulated by the participants’ age.

We then ran an additional pre-registered analysis comparing the children in Experiment 6 with those in Experiment 5. Interestingly, relative to four to seven-year-olds, eight to ten-year-olds were not just more informative when speaking to children (*β* = 0.340, *p* < 0.001), but also when speaking with adults (*β* = 0.137, *p* < 0.001). In a post-hoc exploratory analysis, we compared mixed effect models with and without age as a factor and found a significant effect of age (*χ*^2^ = 121.95, Df = 1, *p* < 0.001); see Figure 2d.

Note that in Experiments 5 and 6 we referred to the adult as a “busy adult”. In principle therefore, rather than adding redundant information for the younger children, participants may have selectively eliminated the adjectives for the “busy” adults. (Alternatively, participants might have added more redundancy than they would have otherwise to compensate for the “business”.) Future research might see if the results replicate without the adjective “busy”. However, this account seems unlikely given that child speakers (across both Experiments 5 and 6) provided fewer redundant color adjectives overall (independent of audience) than the adult speakers did in Experiments 1–3. Indeed, a post-hoc exploratory analysis between all adults (in Experiments 1–3) and all children (in Experiments 5 and 6) showed that children produced far fewer redundant color adjectives overall (*t* = 38.93, *p* < 0.001). Indeed, comparing Spanish speakers addressing adults (in Experiment 2) with eight to ten-year-olds addressing two-year-olds (in Experiment 5) we found that children produced fewer redundant color adjectives in the condition when they were *most* likely to produce them at all than adults did in the condition when they produced them *least* often (*t* = 4.56, *p* < 0.001).

These analyses are exploratory and should be replicated in a pre-registered design. However, they suggest that in addition to any developmental change in children’s sensitivity to audience design and younger children’s delayed information processing, there may also be a broader developmental change in the extent to which children recognize the value of color cues as informative for visual search. A large body of work has suggested tight links between the informativity of color words and their evolution and production cross-linguistically (Conway et al., 2020; Gibson et al., 2017; Lindsey et al., 2015; Lucy & Shweder, 1979; Regier et al., 2015; Rohde & Rubio-Fernandez, 2022; Steels & Belpaeme, 2005; Stefflre et al., 1966). However, we believe this is the first study to suggest that an understanding of the informativity of color words may emerge only gradually over development.

#### Exploratory Meta-Analysis.

Experiments 3, 5, and 6 were underpowered to include subjects as a random intercept in the model. However, we can compare participants (whether adults, children, parents, non-parents, English or Spanish speakers) who saw identical stimuli (the 8-shape arrays) in the adult and child-directed conditions across all five experiments. This meta-analysis shows a significant effect of partner condition (*β* = 21.59, *p* < 0.001). Again, this is a post-hoc exploratory analysis and future work should replicate these findings but the current results are consistent with the hypothesis that speakers are sensitive to listeners’ on-line incremental processing and provide more informative descriptions for children than adults.

## GENERAL DISCUSSION

Across six experiments, we found that English-speaking adults use more redundant color adjectives when speaking to children than adults. We found no evidence that this effect was due to experience talking to young children; parents and non-parents were equally likely to be over-informative when talking to children. This was also true of Spanish speaking adults, even though Spanish speakers were less likely to use redundant color adjectives than English speakers overall (replicating previous work on differences in redundant color adjective use in speakers of prenominal and postnominal modifying languages). Finally, children themselves produced more redundant color adjectives for children than adults. Collectively, these results are consistent with the Incremental Collaborative Efficiency Hypothesis (Jara-Ettinger & Rubio-Fernandez, 2022; Rubio-Fernandez et al., 2021). Adults appear to be intuitively sensitive to children’s relatively slow online processing and spontaneously compensate by building more redundancy into their communication.

Of course, there are other reasons besides sensitivity to incremental processing that might cause speakers to produce more redundant color adjectives when communicating with children than adults. Speakers might believe for instance that children are more distractible than adults and provide more redundant cues to compensate for the possibility that children are less likely to be attending overall. Alternatively, speakers might believe that color is salient and appealing to children and that providing color adjectives might support children’s engagement with the task. Finally, adults might generally provide more descriptive language in talking to children than adults. Critically however, the results of Experiment 4 suggest that none of these is the case. When all the shapes were the same color, thus the use of color adjectives would not improve the efficiency of visual search, speakers were no more likely to use redundant color adjectives with children than adults. This suggests that speakers are sensitive to children’s slower online processing and specifically use more redundant color adjectives with children in contexts when doing so could facilitate children’s ability to identify the target.

There are also a few important caveats on this work. First, although the results of both the subjects-effect analysis in Experiment 2 and the post-hoc meta-analysis are encouraging, future work should replicate these results with larger sample sizes to ensure that the effects are robust. Second, in these experiments, we took the perhaps unusual step of running an “offline” study (no listener was actually present) to study speakers’ sensitivity to listeners’ online incremental processing. This accomplished a number of pragmatic ends. It controlled for individual differences in listeners’ attention and for fluctuations in attention over the course of the experiment. It also halved the number of participants required (by eliminating the need to recruit a listener for every speaker) and eliminated interpersonal confounds based on the particular social dynamic between each speaker and listener. There is reason to suppose that these offline measures sufficed to show speakers’ sensitivity to the difference between adults and children’s online processing. Participants were told that the recordings they were generating would be used as stimuli with actual participants, and the fact that speakers did respond adaptively to the difference between adult and child listeners (and did so only when adding redundant color adjectives would improve efficient search), suggests that they were genuinely simulating the behavior of a future listener.

Nonetheless, we believe in future work it will be valuable to see to what extent these studies replicate in the context of live interactions. One possibility is that speakers might have been *more* sensitive to online processing demands in our study (where the speakers generated the speech acts offline) than they would be in real world situations. In the current experiment, speakers could simulate the ways listeners might respond and did not have to simultaneously produce utterances while tracking an adult or child’s actual fluctuating attention. However, is also possible of course, that speakers’ sensitivity to their partners’ online incremental processing may have been attenuated by the online nature of these experiments where no actual listener was present. Previous work has suggested that sharing a physical environment (“co-presence”) affects communication (i.e., as when listeners’ use speakers’ eye gaze to disambiguate referents; Hanna & Brennan, 2007; Richardson & Dale, 2005); speakers may show a greater sensitivity to their listeners’ processing—and be even more likely to generate redundant color adjectives—in more realistic, interpersonal interactions (see Rubio-Fernández, 2016). These contexts may also be more likely to show effects of array density on speakers’ tendency to produce redundant adjectives.

We also note that we did not screen participants for color blindness in this study; however, relatively few of our study relied on red/green contrasts (the most common form of color blindness) and the relatively low incidence of color blindness in the population means this is unlikely to affect the results. Finally, the current study looked only at speakers’ tendency to provide redundant information—not its impact on learners. Prior work suggests that redundant color adjectives are indeed helpful to adults in visual search tasks (Rubio-Fernandez, 2020). Future work might look at whether the same is true for children.

This study raises many other questions for future investigation. We showed that adults are more over informative when they believe they are speaking to a young child but how do adults titrate their informativity as children grow up? Do speakers gradually wean children from additive information or are there more abrupt transitions? And given that adults with minimal experience and children themselves provide compensatory information to young children, how do people learn to adjust their communication appropriately? Finally, although we know redundant cues facilitate visual search in adults, we do not know to what extent young children benefit. In the current study, both the adult and child listeners were fictitious. Future work might directly test the impact of redundant color adjective use on the efficiency of children’s visual search.

If it turns out that the benefits to children are substantial, then the communicative pressures that support the evolution and production of color words (Conway et al., 2020; Gibson et al., 2017) might have been enhanced by their impact on the youngest learners. Indeed, children’s own production of redundant color adjectives first approaches adult-like levels when children are speaking to children younger than themselves. Given that across languages, and independent of their experience with children, people consistently and selectively generate redundant color adjectives when speaking to young children, it would be intriguing to speculate that much of human sensitivity to incremental online processing might be driven by the support it provides to the very young.

## ACKNOWLEDGMENTS

We want to thank the adults and families who participated in these studies, Karolina Cabrera and Kiera Parece for help with the Spanish language task; the MIT BCS Research Scholars Program, and the NSF (142921, 1823919, 2209756), Siegel Family Foundation, and Halis Family Foundation.

## FUNDING INFORMATION

NSF (142921, 1823919, 2209756), Siegel Family Foundation, and Halis Family Foundation.

## AUTHOR CONTRIBUTIONS

M.T.: Conceptualization; Data curation; Formal analysis; Investigation; Methodology; Project administration; Software; Supervision; Validation; Visualization; Writing – original draft. L.S.: Conceptualization; Funding acquisition; Methodology; Project administration; Resources; Supervision; Writing – original draft.

## CODE AND DATA AVAILABILITY STATEMENT

All code and analyses to reproduce analyses in paper can be found here at this OSF link: https://osf.io/7fysr.

## Notes

^1^ We were unable to test subject effects in this or the following experiments because the necessary increase in participant costs became prohibitive. As discussed in Experiment 5, the fact that the results are consistent across all five experiments suggests that the results are robust but future research should further investigate this claim.^2^ We are grateful to an anonymous reviewer for suggestions that led to the design of Experiment 4.
